# Women Leaders Transcending the Demands of Covid-19: A Positive Psychology 2.0 Perspective

**DOI:** 10.3389/fpsyg.2021.647658

**Published:** 2021-06-03

**Authors:** Claude-Hélène Mayer, Michelle S. May

**Affiliations:** ^1^Department of Industrial Psychology and People Management, University of Johannesburg, Johannesburg, South Africa; ^2^Europa Universität Viadrina, Frankfurt Oder, Germany; ^3^Department of Industrial and Organisational Psychology, University of South Africa, Pretoria, South Africa

**Keywords:** qualitative, hermeneutic phenomenology, TSAI, Ardern, Merkel, speeches

## Abstract

This article explores selected speeches of three global women leaders during the time of the Covid-19 pandemic from positive psychology perspectives. It focuses on speeches to address and manage the pandemic of global women leaders, such as Angela Merkel (Germany), Jacinda Ardern (New Zealand), and Tsai Ing-Wen (Taiwan). This study explores the question what global women leaders' leadership actions and responses are and how they address their nations with regard to the four pillars of PP2.0 and the PURE model during Covid-19. The study uses a post-modernist qualitative research design. It is anchored in the hermeneutical-phenomenological research paradigm, using leadership theories and PP2.0 as a lens to explore and understand their strengths with regard to the Covid-19 pandemic. The authors use thematic analysis to analyse the selected speeches made by the three women leaders at the onset of the pandemic in Germany, New Zealand and Taiwan. The study contributes to improve the understanding of global women leadership during Covid-19. Conclusions are drawn. Recommendations will be made accordingly.

## Introduction

During Covid-19, the topic of the leadership of women in political roles and leaders of countries has gained interest and sparked new research (Freizer, 2020). Maclean (2020) has pointed out that in particular women leaders, such as Jacinda Ardern Jacinda Ardern of New Zealand, Tsai Ing-Wen of Taiwan and Angela Merkel of Germany have all been praised how they have handled the coronavirus pandemic and they “have been praised for demonstrating care, empathy and collaborative approach.” Even Merkel, who is known for her information and fact-based political approach starts showing emotions and talks in emotional ways to the nation during Covid-19 (Emundts, 2020). Maclean (2020) points out that these attributes have traditionally been ascribed as “feminine” and have led to transparent and accountable leadership during the time of panic and mass confusion. However, the author also highlights that this “feminine” ascriptions need to be viewed as dangerous since they might reinforce stereotypes of women leadership.

This article focuses on women leadership during the Covid-19 pandemic in the light of Positive Psychology (PP) with special regard to Positive Psychology Wave II (PP2.0). PP is the scientific study of positive experiences and positive individual traits as well as its development (Peterson and Seligman, 2004). It deals with fostering positive emotions and creating new character strengths which can directly or indirectly alleviate suffering and undo its root causes (Nolen-Hoeksema et al., 2005). Paul Wong, as the pioneer of the PP2.0 movement criticized with regard to the first wave of positive psychology its single focus on positive aspects on life (Wong, 2019, 2020a,b). Therefore, PP2.0 essentially integrates the negative and the positive aspects in life and life's ambivalent nature (Wong, 2020a).

Henrich et al. (2010) have criticized that most PP research has been based on “WEIRD” sampling, taken Western, Educated, Industrialized, Rich and Democratic participants into consideration. For a long time, PP has been assumed to be a universal concept, however Lomas (2016) has pointed out that cultural differences and nuances need to be taken into consideration.

A recent article of Waters et al. (2021) has pointed out that PP factors can play a buffering tole against mental illness during the pandemic. They further highlight that PP factors also bolster mental health during Covid-19, thereby strengthening future mental health. The authors emphasize nine extraordinary factors which support individuals during the pandemic, such as meaning, coping, self-compassion, courage, gratitude, character strengths, positive emotions, positive interpersonal processes and high-quality connections. Finally, the authors call for an increase in PP research during the pandemic to investigate factors which sustain and strengthening mental health and well-being (Waters et al., 2021).

This research focuses on women leaders from different cultural backgrounds. It is the case that several of them come from Western, educated, industrialized, rich and democratic background, but not all of them. This research does not only focus on the participants from WEIRED samples, but takes leaders from different countries and contexts into account. Further, the authors primarily focus on women leaders. It can be highlighted that world leaders' experience increasing demands for containment and safety from their followers in the context of the covid-19 pandemic (Looi, 2020), as well as for the reimagining of inclusive political processes (Maclean, 2020). It seems that the women world leaders have dealt with the pandemic much more effectively than their male counterparts (Freizer, 2020; Maclean, 2020), due to, for example, an earlier lockdown and a more citizen-centered approach (Barry, 2020). In order to understand this apparent leadership success on the part of female world leaders, the research question is posed: What aspects of PP2.0 contribute to women political leaders' approach to combat Covid-19?

By responding to the question, the authors will primarily focus on the current literature of women in political leadership in Covid-19, selected aspects of PP2.0 and the ability of women leaders to transform pain, suffering, fear and despair into meaningful actions within times of Covid-19.

## (Women) Political Leadership and Covid-19

Morrell and Hartley (2006) identifies four kinds of leadership theories namely, trait, contingency, situational and constitutive approaches. These theories focus on either the individual or the context or the interaction between the two. Political leadership, a subtype of human social leadership, is a socially constructed and a multidimensional phenomenon signifying the interdependence of the leaders and their contexts marked by leader-follower relations, task structure and power positions (Masciulli et al., 2016) in a turbulent context. Political leadership also ensures political and social interconnection between, public administration, business and the rule of law in national systems (Ali et al., 2017). Thus, political leaders have different roles with concomitant responsibilities dictated by (turbulent) contexts resulting in leadership being socially constituted and relationally constricted (Morrell and Hartley, 2006).

Political leaders are those who hold formal political authority, acquired through democratic election in a democratic society (Morrell and Hartley, 2006, p. 484, 485). Political leaders using collaboration to charisma and integrity take up different roles at different levels, representing and articulating the view of diverse groups within different contexts (Ali et al., 2017). These contexts can be reasonably stable or have varying degrees of turbulence such as that marked by the current complex global uncertainty and crisis due to the COVID-19 pandemic (Morrell and Hartley, 2006; De Clercy and Ferguson, 2016). Political leaders are centrally involved in defining and interpreting the problems confronting the political community, designing innovative and creative solutions through the cocreation and co-production with citizens and organized stakeholders (Torfing and Sorenson, 2019), ensuring political and popular support for the implementation of these solutions and communicating these solutions effectively (Tucker, 1995; Masciulli et al., 2016). Thus, interactive political leadership refers to the cocreation and co-production of public value outcomes by political leadership through deliberately initiating, orchestrating and participating in efforts to harness the knowledge, ideas and resources of citizens and organized stakeholders in problem-focused processes including precarious contexts (De Clercy and Ferguson, 2016), such as the COVID-19 pandemic. However, these collaborative efforts do not only occur with knowledgeable, real and authentic citizens, but also with those considered to be unreasonable, indifferent and ignorant (Lees-Marshment and Smolović Jones, 2018).

In leader-follower relationship, the leader utilizes certain personal characteristics: inherent qualities, socialized habits, learned skills; intelligence of various types, including especially emotional intelligence and contextual intelligence, including social insight; but also power-wielding, organizational and communication skills (Masciulli et al., 2016, p. 15). Thus, a crucial important aspect of interactive is leaders deliberately using their interpersonal skills to systematically mobilize widespread collaboration amongst organized stakeholders and citizens, public and private actors in formulating and implementing innovative and creative solutions to unruly and wicked problems in a political context marked by government and business policies (Ali et al., 2017; Lees-Marshment and Smolović Jones, 2018). Through IPL, responsive and responsible political leaders, create multifaceted stories about wicked problems for the communities, engaging multiple voices, perspectives, experiences and observations in creating and jointly owning the solutions to these problems through active citizenship (Torfing and Sorenson, 2019). Thus, political leadership can be conceptualized in relational, collaborative and formal terms where the political leaders should demonstrate their openness to new ideas, changing and learning (Masciulli et al., 2016), their ability to facilitate dialogue, agency and consent of others (Morrell and Hartley, 2006), as well displaying mastery, strength, competence and assertiveness as required by the context (Lees-Marshment and Smolović Jones, 2018).

Thus, political leadership and the interactive relationship between the leader and follower is socially constituted and relationally constructed (Morrell and Hartley, 2006). Consequently, the political leader can decide which agency is required and the followers ultimately decide which behaviors are considered effective detailing an interdependence between the leader and followers (Morrell and Hartley, 2006). Since 2001 political leadership has been studied as crisis leadership (Körösényi et al., 2016; Torfing and Sorenson, 2019). During times of crises, political leadership should produce a culture of strong and clear vision using different strengths to pursue this vision, including authorizing and mobilizing their followers in constructing and implementing this vision to ensure the sustainable welfare of all (Ali et al., 2017). Political leaders, during crisis, could either be reactive or interpret the crisis to determine a strong vision, subsequently using the creative interaction between the leader and the follower in determining and implementing solutions. Through this relationship marked by creativity the leader effectively communicates based on the interpretation of the crisis a strong vision to be achieved by the leader, the leadership team, as well as the followers to ensure the sustainable welfare of all (Körösényi et al., 2016; Torfing and Sorenson, 2019).

Women remain underrepresented in and the study of women leadership is neglected or treated differently in political leadership. Women leaders' decision-making capacity and consequently their effectiveness as leaders, are judged based on conceptions about their motherly role, them being emotional and their physical attributes unrelated to their leadership capacity (Sjoberg, 2016). Gendered leadership based on assumed group characteristics, perpetuates the valuing of male and masculine characteristics, such as strength, power, autonomy, independence and rationality, typically, while devaluing female and feminine characteristics, such as emotionality, passivity, dependence, marginalization and weakness. However, individual women (and men) experience gendering and the processes by which gendering operate differently based on their diversity. Furthermore, men are associated with the public sphere (work politics and public life) and women are associated with the private sphere (motherhood, household and the bedroom). These conceptions of gendered leadership associating good leadership with male characteristics and weak leadership with female characteristics are further reinforced though gender tropes which denotes gender norms and stereotypes which reinforces existing gendered leadership (Sjoberg, 2016; Sykes, 2016).

According to Johnson and Williams (2020) COVID-19 changed the division between the private and public spheres of politics allowing the valuation of feminine characteristics of women political leaders. The coronavirus pandemic has mobilized political leaders to use protective masculinity and more rarely, protective femininity in their endeavors to ensure the sustainable safety and security of citizens (Johnson and Williams, 2020). Consequently (women), political leaders can more easily access the gendered stereotypes of protective femininity marked by caring and empathy associated with women's role in the family and in this way probably facilitate their followers experience of being looked after. Several women leaders have received recognition as voices of reasons and influence during the coronavirus pandemic. The maternal status of the female political leaders seems to be foregrounded for them to blend science “epidemiology... with empathy, laws with mom jokes” and, after caring for their children, “empathizing with citizens' anxieties” (Johnson and Williams, 2020). As women political leaders harness the gendered (female) leadership to their advantage, the (women) political leaders also interpret the crisis in a particular way for their followers, in order to generate shared solutions and relevant changes and its concomitant implementation (De Clercy and Ferguson, 2016; Johnson and Williams, 2020).

Women political leaders have shown strength in proposing and implementing challenging strategies, while harnessing (supposedly feminine) characteristics such as caring empathy and compassion. Merkel, also nicknamed Mommy, expressed compassion and understanding for her followers as she asked she stated challenging interventions to curb the spread of COVID-19. During a speech outlining difficult strategies, Adern has apologized for her appearance due to putting her toddler to bed. Furthermore, Merkel, Adern and Tsai Ing-wen were praised for their “effective messaging and decisive action,” during the pandemic (Johnson and Williams, 2020). It is evident, several women political leaders have shown effective leadership through their rapid and aggressive response, as well as strong adaptive leadership marked by cooperation amongst themselves, public and private sector stakeholders and citizens. Through this effective woman political leadership, a better and more resilient post-pandemic world can possibly be developed (Olayinka, 2020). We suggest, following on Johnson and Williams (2020), that these political leaders have demonstrated the value of feminine protective characteristics, i.e., caring, empathy, compassion and creativity, as part of their wider leadership toolkit.

## Positive Psychology 2.0

Lomas and Ivtzan (2016) emphasize that humans need to appreciate the dialectical nature of well-being, thereby dealing with negative aspects of suffering, despair, as well as negative emotions, as well as the positive aspects. In PP2.0 approaches, the “dark” side has to be explored, accepted and finally transformed toward a meaningful integration and finally the “lightful” aspect of the experienced circumstances needs to be developed out of the conscious work with and transformation toward the pain and the suffering (Van Tongeren and Showalter Van Tongeren, 2020; Wong, 2020a,b). The PP2.0 approach is therefore a more detailed and nuanced approach how to see the positive developing from the conscious experience of the negative (Wong, 2011). Further, the PP2.0 approach deals with life and life experiences as an existential concept, taking existentialistic aspects into account, always referring back to the concept of meaning and contextual meaningfulness and the question what meaning can be derived from suffering, from pain, as well as from positive experiences of happiness or positive well-being (Lomas, 2016; Wong, 2020a,b).

During the past years, Wong (2011) has developed the concept of PP2.0 and refined its pillars. Thereby, PP2.0 expands the first wave of positive psychology by focusing on transforming suffering and pain into individual growth (Wong, 2021a,b). Wong's (2020c; 2021a) PP2.0 concept includes the self-transcendence model of flourishing which is based on the ideas of existential positive psychology. Main pillars of PP2.0 are: Virtue (including the moral imperative), meaning (emphasizing the centrality of meaning), resilience (highlighting the intrinsic human capacity of resilience) and well-being (integrating the universal human yearning for happiness and a vision of an improved future).

Developing human strengths and civil **virtues** is in the focus of PP (Peterson and Seligman, 2004). McCullough and Snyder (2000, p. 1) define virtues as: “psychological process that consistently enables a person to think and act so as to yield benefits to him or herself and society.” Peterson and Seligman (2004, p. 29–30) distinguish six broad categories of virtues – which are further broken down into twenty-six characters: wisdom, courage, humanity, justice, temperance, and transcendence. Virtues impact on transcendent experiences and optimal human functioning (Mayer and May, 2019, p. 157) and can support the overcoming of negative or challenging life events. The virtue of transcendence includes the character strengths, such as: appreciation of beauty, gratitude, hope, humor, and spirituality. Wong (2021b) has pointed out that PP2.0 is extremely important during the pandemic, since PP2.0 integrates suffering and pain to strive toward individual development and growth, it addresses existential threats and advances global well-being - by being human-centered - and finally it provides researchers with a dialectic dual system strategy which can build the bases for research and psychological interventions. In general, Wong (2021b) points out that PP2.0 is part of a spiritual vision for global well-being through self-transcendence.

Additionally, the virtue of transcendence adds **meaning** to a person's life (Peterson and Seligman, 2004). It also helps to overcome pain and suffering and fosters the will to live and, in this case, to overcome the pandemic. Meaning is central to the development of happiness and a good life (Peterson and Seligman, 2004; Wong, 2015, 2020a,b). Wong (2012) emphasis that the PURE-model of the structure and function of meaning is important for the analysis of experiences and behavior, as explained in the following:

1. **P**urpose involves the overall direction, life goals, and core values. It provides the framework of daily deliberations and decisions. Further, it includes existential values and emphasizes the reflection of what priorities in life and matters. Purpose connects the individuals to a higher force and to the devotional.
2. In the center of **U**nderstanding is the concept of self-identity, but also the comprehensibility of how the world works and therefore understanding relates to the individuals, the self-understanding and self-knowledge, but also the understanding of the systems and the environment which consists around a person (Wong, 2012, p. 10, 11). To develop an in-depth understanding, individuals need to develop a sense of coherence, in-depth understanding of life and death, but not only from a knowledge perspective, but rather from a self-reflection perspective. Thereby, the individual needs to understand his/her place in life to dive deeper into the complexity of life and environmental connections. Finally, to lead a meaningful life, an individual does need self-knowledge, self-reflection and self-acceptance.
3. **R**esponsibility is important when acting and reacting and when developing behaviors (Wong, 2012). Responsible actions are widely connected to concepts of ethics and ethical behavior, success as well as well-being of self and others.
4. Finally, **E**njoyment and evaluation refer to positive feelings and their connections to good actions (Wong, 2012) which should usually be based on purpose, understanding and responsibility. If a person is dissatisfied with the actions and misses enjoyment, the purpose, understanding and responsibilities need to be re-evaluated and the recognition of potential negative aspects can then lead the individual or group toward positive change.

These four pillars need to be enhanced for personal self-transformation, while the individual has to constantly work on the management and transformation of the inherent suffering, pain and despair in life. When this is the case, then, Ivtzan et al. (2016) emphasize, individuals will grow, gain deeper insights, healing and transformation. However, the concept of PP2.0 does not only focus on the self-development, but also on the improvement of life for all people and humankind (Wong, 2011). This becomes particularly important in Covid-19 times where women leaders have been highlighted to lead in the interest of the people and their countries mainly (WEF, 2020). However, the leadership of women and the virtues and meaning leadership is based upon, has hardly been researched in depth in the speeches of contemporary women leaders in political roles – nor has their leadership been reflected upon from PP2.0 perspectives. Thereby, Wong (2011) suggests that PP2.0 needs to take positive and negative emotions and experiences into account to develop holistically. According to Wong (2020a,b), this is then rather a balanced model which integrates strengths and weaknesses and responds well to the complexity of life. Thereby, the eminent suffering and pain in life and the enhancement of happiness need both be accepted as integrated parts of life and therefore need to be addressed as interdependent variables (Wong, 2011; Wong and Worth, 2017).

In 2020, a third wave of positive psychology has been announced (Lomas et al., 2020). However, this wave is not discussed in this paper further, since the authors focus on exploring aspects of PP2.0 in more depths.

## Research Methodology

In the following, the research paradigm, sampling, researcher roles, data collection, analysis and interpretation will be described.

### Research Paradigm

This study uses a post-modernist qualitative research design (Creswell, 2013). It is anchored in the hermeneutical-phenomenological research (Dilthey, 2002). paradigm to explore and understand the perspectives and actions of women leaders with regard to the Covid-19 pandemic, responding particularly to the research question what the dynamics of their leadership actions and responses are when dealing with Covid-19.

The hermeneutical paradigm is viewed by Mullen (2019) as being similar to gestalt and thereby, as described by Atwood and Stolorow (1984), as a methodology approaching and accessing the knowledge of the whole by constituting a study of its parts. Hermeneutics explore a deeper “Verstehen” and thereby aims to create meaning which is implemented in the engagement with the entire text (Kripal, 2007). With regard to this article, the authors aim at understanding the dynamics of the subjective experiences of the women leaders within their social, cultural and global contexts (Clarke and Hogget, 2009; Creswell, 2013; Hassan and Ghauri, 2014).

### Sample

The sample is comprised of selected women leaders who are well established in their countries', as well in the world's political scene, reacting to Covid-19 in the beginning of the year 2020.

- Angela Merkel -Germany
- Jacinda Ardern – New Zealand
- Tsai Ing-Wen – Taiwan

The criteria of selection for the women leaders included in this analytical research are as follows:

- They are female leaders.
- They have a world leading position in the political arena their country during the Covid-19 pandemic outbreak, such as chancellor or prime minister.
- They have held a speech at the beginning of the Covid-19 outbreak which is available for analysis.

### The Researcher Roles

Both researchers are working in the field of Industrial and Organizational Psychology in South Africa. One is a clinical psychologist with specialization in systems psychodynamics while the second researcher is a research psychologist and a systemic therapist. Both researchers have been working on issues regarding women in leadership and have both held leadership positions themselves.

### Data Collection, Analysis and Interpretation

The data used for this article were collected from open-access public sources. For each women leader the initial public speech to their country about how to address the Covid-19 pandemic was used as source of analysis. Three information-rich speeches were analyzed and interpreted in detail. Data were ordered, organized and reorganized with regard to certain patterns, categories and descriptive units (Patton, 2002). Through the hermeneutical analysis of the text (Dilthey, 2002), the researchers got into a hermeneutic conversation with the texts and thereby contributed to creating meaning while explaining descriptive patterns and dimensions (Patton, 2002).

The analysis was created by using the model of Terre Blanche and Durrheim (2002), seeing the process of data analysis as back and forth movement, in this case referring to hermeneutic phenomenology. The analysis of the data involved the immersion/crystalisation style which entailed *becoming thoroughly familiar with the phenomenon, carefully reflecting on and then writing an interpretation* (Terre Blanche and Durrheim, 2002, p. 140). The interpretive analysis became a substantial part of building the hermeneutic circle which is viewed as the process of interpretation of meaning (Dilthey, 2002). The steps followed were the ones indicated by researchers, such as Miles and Huberman (1994), Terre Blanche and Durrheim (2002), Terre Blanche et al. (2006a), and Yin (2009, 2014):

1. Familiarization and Immersion

The authors familiarized themselves with the women leaders and global politics and their initial speeches referring to Covid-19.

2. Inducing themes

Naturally underlying data in terms of themes and categroies were determined as principles. And sub-themes were developed (Terre Blanche et al., 2006a).

3. Coding/categorizing into meanings

While analyzing the data, codes were developed (Miles and Huberman, 1994). Codes were further clustered in relation to other clusters as well as the themes clusters (Miles and Huberman, 1994; Terre Blanche et al., 2006b).

4. Generating and elaboration of themes and patterns;

By employing “constant comparison,” recurring phrases and patterns were identified and categorized and clustered with regard to the themes and their thematic similarity. Themes were then elaborated and included dynamic contradictory information which again led toward sub-themes and sub-issues.

5. Interpretation and checking

Finally, data in terms of themes, categories, and were reconsidered and interpreted and the questions regarding of the meaning of the data through the eyes of the researchers were addressed (Miles and Huberman, 1994).

6. Reporting of data

Finally, the data analyzed and interpreted were integrated with the literature and the data were written up as presented in the findings and discussion section.

### Qualitative Quality Criteria and Ethical Considerations

Credibility was ensured through triangulation and using different sources, methods and theoretical approaches (Creswell and Plano Clark, 2011). Confirmability and transferability of data (Creswell, 2013) were promoted through intersubjective validation processes of the two researchers and the use of established theories and methods (Yin, 2009), while rigor was promoted through thick descriptions and transparent processes. The study further provides an in-depth insight into the data, the findings and the topic as a whole, but does not provide generalisability (Lincoln and Guba, 1985; Creswell, 2013). Ethical approval was provided by the European University in Germany university in Frankfurt (Oder). Data were only taken from the public domains and did not interfere with the private spheres of politicians.

## Findings and Discussion

In the following, the findings regarding global women leaders, their leadership, PP2.0 and the PURE-Model will be presented.

### Women Leadership and Covid-19

Using the leader-follower relationship, specific aspects of women political leadership will be highlighted:

#### Firm, Clear Communication of Citizen's Responsibility

All three the women political leaders firmly called their citizens to action, responsibility, contribution and co-operation against the pandemic through their initial speeches. These leaders used science and economic resources, as well as the human resources as a means of working toward preventing the catastrophic impact of the pandemic. Merkel (2020) connected her firm requests to consultations with the scientific community in Germany and what has been experienced internationally. Ardern (2020) used the international experience of the exponential growth of the pandemic to cushion her instructions to citizens. Ing-Wen (2020) used the scientific capabilities of the country as a vehicle to call the citizens to responsibility and co-operation.

The three leaders showed a clear plan on the part of government, as well as clear directions to different stakeholders and citizens in the endeavors to deal with the pandemic. Merkel (2020) without mincing her words announced the lockdown and then clearly and firmly outlines the plan for different stakeholders, including citizens in their struggle against the pandemic. She clearly and firmly communicates her expectations of the citizens using her and their personal experiences to show their capability to deal with challenges, as well as care for others. She clearly outlines the subtasks and the boundaries that for these expectations in their roles as citizens. She also firmly highlights the tasks, boundaries and authority of other stakeholders such as, government officers, frontline workers, staff of shops selling essential goods, and other stakeholders. Ardern (2020) also clearly and firmly outlines the boundaries, authority, roles and tasks of different stakeholders including the citizen during the lockdown to curb the pandemic. She clearly communicates, explains, and shares aspects of the plan and in this way engages the citizens so that they can take ownership of the envisaged challenging plan to deal with the pandemic. She uses phrases such as, “I also said we should all be prepared to move quickly,” “Now I want to share with you …,” “To be absolutely clear we are now asking...,” “we are all now preparing …” to prepare the citizens for a difficult strategy that they should take ownership of.

Ing-Wen (2020) acknowledges and seems to speak with pride of their position within and contribution to the fight against COVID-19 in the global context. She warns about how the conditions in other countries will have a direct impact on the spread of the virus in Taiwan. She further emphasizes the need to contribute to the international efforts by pooling their capabilities to overcome the challenges brought by COVID-19. She emphatically echoes to the Taiwanese citizens the need for contributing responsibly through their capabilities to the international community. Perhaps in showing their international impact, she is also bringing a sense of how they can help each other nationally, at community level, as well as family level. In other word, if the Taiwanese can contribute internationally, they definitely can with more ease contribute nationally and on family/individual level to stop the spread of the virus. Although Ing-Wen's (2020) focus is on the international community, she also outlines a plan for citizens to bring their capabilities to provide masks, support for pharmaceuticals, as well as technology support nationally. Thus, by asking Taiwanese citizens to use their efforts to provide support through surplus in the international arena, Tsai Ing-Wen ensured that they first provide the support to national stakeholders and particularly their fellow citizens.

#### A Firm, Clear Request Through Own Humanity

Merkel (2020) in her speech specifically declares and reveals her humanness through her relationship with her followers. She starts by lamenting how she is remained of other challenging, maybe even traumatic time as a member of the German community. She declares her fundamental belief that everyone's life counts and that she cares for the lives of others through her efforts to preserve all life. She shows deep gratitude for those who may think that their jobs are not valued, as well as for those who risk their lives at the forefront of dealing with the ill. Showing her willingness proactively to work, as well as use her personal and national resources to slowdown the spread of the pandemic. She also expresses her understanding of how difficult social distancing as a way of caring for others are by acknowledging her need to show affection through physical closeness or touch. Ardern (2020) and Ing-Wen (2020) also reveals and declares their humanity in the context of her relational interaction with her followers.

#### A Firm, Clear Request Through Shared Humanity

Merkel (2020) demonstrates that she understands the extend of the sacrifice she is demanding of and that this sacrifice may become even harder on the German citizens through the severity of the lockdown. However, she reminds them of their communal responsibility. She calls for unity and solidarity from all in this difficult time. She also reminds the citizens of their responsibility in not only caring for each other, but also saving each other's lives. In this way highlighting their agency, i.e., “we are not condemned to passively accept the spread of the virus,” in preventing the exponential spread of the virus. Merkel (2020) also invites citizens to use their resources in a creative way to deal with the demands of the lockdown: There are already many creative forms that defy the virus and its social consequences. Already there are grandchildren who are recording a podcast for their grandparents, so they won't be lonely. Merkel (2020) also show the citizens that have creatively maintained contact with others despite social distancing: “We now hear about wonderful examples of neighborhood help for the elderly who cannot go shopping themselves.” Then she implores them, to continue with their community commitment/awareness, to use and develop creative ways of enhancing their social contact despite preserving social distancing. In some level she cleverly say to the citizens, I have noticed that you have been doing what I am asking you now.

Ardern (2020) announces with the lockdown plan a 48-h transition period for different stakeholders to plan for the hard lockdown marked by self-isolation. She clearly communicates the task for different role players during this transition period: “So over the next 48 h every workplace must implement alternative ways of working” and “Essential services will need to put in place alternative ways of working that ensure physical distancing of staff of 2 meters,” She clearly shares information that will help citizens to implement the plan for a successful lockdown. In this way she clearly communicates the tasks and the boundaries for the new reality to ensure that citizens are fully authorized in their different roles take up the responsibility required to successfully battle the pandemic.

Ing-Wen's (2020) cushions her expectations of her citizens in their responsibility to benefit others internationally and nationally through a collective effort of support. She reminds the citizens of their international duty and in this way reminds them of their national duty. To extend this idea she reminds the citizens of the countless acts of bravery and sacrifice by medical workers around the world – risking their lives to protect the lives of others. Perhaps in this way she asked he citizens to engage their bravery and sacrifice for the greater good. She also implores citizen to share with those who are struggling, besides sharing with those who are at the forefront of dealing with COVID-19 patients. This outcry to share, may also suggest an outcry for care, compassion and empathy for fellow-citizen and fellow-humans.

These women political leaders called their citizens to take up responsibility, act courageously, make a contribution, have compassion and empathy for others, possibly engaged the citizens of these three countries as relational beings through the leader-follower relationship they have with their citizens. As accentuated by Ing-Wen's (2020) they should not just talk the talk, but walk the talk, i.e., “We want everyone to not only see that “Taiwan can help,” but that “Taiwan is helping.”

Based on this discussion Table 1 has been constructed.

**Table 1:** Global women leaders engaging their citizens as relational being (Authors own construction).

**Political leadership**	**Angela Merkel Germany**	**Jacinda Ardern New Zealand**	**Tsai Ing-Wen Taiwan**
Citizen responsibility	•country that depends so much on our joint solidarity. •what you do is tremendous •learn from the international community	•through decisive action, and through working together •Act now, or risk the virus taking hold •we are all now preparing to go into self-isolation as a nation	•Assistance to the international community •‘Taiwan is helping.’ •As we face the threat of COVID-19, our entire country has worked together
Own humanity	•I firmly believe that we will succeed in this task •for [me] whom freedom of travel and movement was a hard-won •and I thank you for it with all my heart	•I understand that self-isolation is a daunting prospect •I do not underestimate what I am asking •Be strong and be kind	•I want to emphasize that this kind of cooperation is possible • [I] have seen countless acts of bravery and sacrifice from medical workers
Shared humanity	•we will succeed in this task if all citizens see it as their task. •And we are a community in which every life and every person counts •everything that could harm not only the individual, but also the community, we must reduce that now	•Everything you will all give up for the next few weeks • [your sacrifice] will literally save lives. Thousands of lives •What we need from you, is support one another	•Over the past months, we established a “national team.” •We will share our domestic electronic quarantine system •we have kept the domestic outbreak well under control

### Global Women Leaders' Speeches in the Light of PP2.0

In the following, the three initial speeches at the beginning of the Covid-19 outbreak are analyzed and discussed in the context of PP2.0.

#### Virtue (Including the Moral Imperative)

Merkel (2020), the German chancellor, in her speech from March 2020 emphasizes that life has changed dramatically with the start of the Covid-19 pandemic and that only common action can help to protect humankind and individuals in the society. Merkel refers to the core values of democracy during the start of the pandemic and her intention to make political decisions which are transparent, understood with the intentions communicated and relatable. She also highlights the impact of every single person in society to defeat the virus and to save lives: “Es kommt auf jeden einzelnen an.” Further values and virtues which she highlights are scientific rigor as basis for decision-making, continuous re-evaluations of the situation based on latest results and she requests trust from the citizen in the actions of the government. Actions of the individuals should focus on a hearty and reason-driving behavior and attitude which takes rules and regulations as being serious interventions. She further points out the importance of the interplay of the individuals and the society as a group. She ends her speech with the imperative the people should take care of themselves and their loved ones: “Passen Sie gut auf sich und Ihre Liebsten auf!”.

Ardern (2020), in her initial speech, choses to refer to fighting the virus through “decisive action and working together,” supporting each other in a kind and strong way while leaving the enforcement of restrictions to the government.

Ing-Wen (2020) highlights that the people of Taiwan must come together to protect the nation and fight the virus. Thereby, Ing-Wen (2020) emphasizes the commitment of Taiwanese people which needs further pro-active efforts, such as tightening the border controls, enhanced testing and containment measures. According to Ing-Wen (2020), fighting the virus needs determination and she asks her citizen to restrain from hostility and blaming. She further on thanks her citizen who comply and cooperate with the government to fight the virus, the frontline workers and the people who come together to fight the virus (Ing-Wen, 2020). Finally, the government will cooperate with others in the world to share openly experience, fight the virus together and contribute to mutual assistance and cooperation.

#### Meaning (Emphasizing the Centrality of Meaning)

With regard to meaning, Merkel (2020) points out that the normality and its ascribed meaning to life is questioned as has never been before: “Unsere Vorstellung von Normalität, von öffentlichem Leben, von sozialen Miteinander - all das wird auf die Probe gestellt wie nie zuvor.”

The core aspects that Merkel (2020) highlights in terms of meaningful actions within the pandemic refers to re-assuring meaning of democratic values through common action and participation of each and every single citizen. The pandemic can be used to re-experience democracy as a political form of shared knowledge and participation which leads to manage the common tasks of the society to defeat the virus. Merkel's message is basic, namely that the task of the Covid-19 pandemic is to secure survival.

For Ardern (2020), the meaning of the Covid-19 measures and the restrictions lies in containing the virus through common action of the government (implementing restrictions) and the citizen (supporting each other, being kind and strong).

Ing-Wen (2020) emphasizes that all the citizen must take care of their health as individuals and the health of their families to protect the nation and that international cooperation is the key. She sees the time of Covid-19 as a call to accelerate international cooperation and to care for the health and well-being of humankind (Ing-Wen, 2020).

#### Resilience (Highlighting the Intrinsic Human Capacity of Resilience)

Merkel (2020) asks for everybody to social distance and is empathetic and understanding that this is a task that is very difficult to manage since humans, in times of emergency. Merkel (2020) further points out that individuals in the German society will find new forms of coming together, meeting and supporting each other. She asks for trust in the decisions of the government which she assures the German society about are built on scientific rigor and updated information. Merkel points out that she is absolutely convinced that Germany will manage this crisis: “Dass wir diese Krise überwinden werden, dessen bin ich vollkommen sicher.” She, however, warns that the numbers of victims of the pandemic are in the hand of each and every single person and is dependent on the degree of disciplined behavior.

Ardern highlights in terms of resilience that people must be strong and enduring, while Ing-Wen (2020) is the only one of the three leaders who highlights that the Taiwanese people are resilient and that they show their resilience in coming together, aiming to fight the virus. She exemplifies this in correspondence with her gratitude for members of the national team coming together to manufacture goods to prevent the spread of Covid-19. At the end of her speech, she again emphasizes the strength and resilience of the Taiwanese people.

#### Well-Being (Integrating the Universal Human Yearning for Happiness and a Vision of an Improved Future)

The impact of the virus, according to Merkel (2020) is mostly a challenge in terms of well-being with regard to the limited social connections and contacts, but she strongly displays a positive vision of a positive future by highlighting the creative solutions to connect across generations and within families through, for example, virtual contacts or measures of using new technologies for podcasts. Well-being, for Merkel (2020), in these times is strongly connected to social connection and support of the elderly, for example, through neighborhood-support. She assures the German society that “…wir werden als Gemeinschaft zeigen, dass wir einander nicht allein lassen.” – we will show each other as a community that we will not leave each other alone.

For the well-being of the people, Ardern (2020) suggests to move quickly and put action plans into action. She highlights that people should plan and find new ways to connect, be kind and be strong together and to unite against the virus, while she emphasizes that it is the responsibility of the government to enforce the restrictions. With that individuals should not interfere to enforce restrictions, but rather “support each other” (Ardern, 2020).

Finally, Tsai Ing-Wen (2020) points out that it is important to “refrain from causing panic and help to spread accurate information.” She emphasizes that the Taiwanese people must “think of others” as well, since the world is facing a global pandemic and Taiwan has to cooperate with international forces.

Based on this discussion Table 2 has been constructed.

**Table 2:** Global women leaders and PP2.0 (Authors own construction).

**Leaders and PP2.0**	**Angela Merkel Germany**	**Jacinda Ardern New Zealand**	**Tsai Ing-Wen Taiwan**
Virtue	•Protection through common action •Democratic values: transparency, open communication and decision-making, relatability •Trust governmental decisions •Act responsibly as individual and societal member •Care for self and others	•Fighting the virus through decisive action and working together •Government will do all to protect citizen and asks citizen to do all they can to protect “us all” •Everybody to play their part to contain the virus to stop the spread •Unite against Covid-19 in a kind and strong way	•Taiwanese people come together to protect Taiwan from the virus •Fighting the virus •Restrain from hostility and blame
Meaning	•Questioned normality and meaning in life •New focus of meaning is on survival and defeating the virus	•Focus is to save lives and contain the virus •Uniting against the virus	•Protecting the health of individuals, their families and thereby the nation
Resilience	•Manage loneliness and trust in finding new forms of connection, particularly in family relationships •Care for self and others through new creative forms of connection	•Act now or risk the virus taking hold •Empathy that restrictions will be daunting for all people •Be kind and strong and let the government enforce the restrictions	•Resilience is shown in the national team's activities to manufacture items needed to contain the spread of the virus •Resilience and strengths are strong in Taiwanese people
Well-being	•Empathy regarding missing social connection and contact •Well-being through social support and action	•Move quickly and put action plans into action •Awareness of socio-economic disruption and anxiety •Well-being through planning keeping in touch, kindness and strongness	•Well-being is increased through helping to share accurate information and refrain from panicking, blaming and hostility.

### Global Women Leaders and the PURE-Model

Focusing on the aspect of meaning, the speeches of the three female world leaders show the following in terms of the PURE-model:

#### Purpose

While Merkel (2020) points out that the nation needs to defeat the virus and survive, Ardern's (2020) purpose is to act fast and contain the virus. Merkel uses rather a term from war times (defeat and survive) while Ardern speaks of containment and saving lives. Lives should be protected through the governmental actions and the actions of the citizen (Ardern, 2020). Ing-Wen (2020) points out that Taiwanese people and the government will fight the virus by working together on all levels and reducing the spread of the virus. For Ing-Wen (2020) it is the time to expand international cooperation, support and openness.

#### Understanding

According to Merkel (2020), the virus and its impact must be understood to make a plan how to approach the defeat of the virus on individual and communal levels while individuals should distance socially and find new and creative ways to connect.

For Ardern (2020) there is an easy way to contain the virus: “Our plan is simple. We can stop the spread by staying at home and reducing contact. Now is the time to act.” She emphasizes that the way how to deal with and understand the spreading of the virus is easy and how to react to it properly to contain it as well. To understand life and death in this way is easy, while the decision to put the nation on lockdown level is a decision not taken lightly (Ardern, 2020). Ardern (2020) repeats several times in her speech that New Zealanders are asked to stay at home and stop interaction with others.

In the Taiwanese context, it is important to share accurate information to understand the situation, to work together and refrain from negative emotional reactions, from panicking, blame and hostility (Ing-Wen, 2020). She thereby refers to positive values which will help the comprehend the situation and what is going on.

#### Responsibility

Merkel (2020) points out that there is a shared responsibility of the government and the citizen to defeat the virus and that all individuals need to support each other and care for each other across generations.

Ardern (2020) emphasizes that quick decision making is needed to act responsibly and develop healthy behavior to save lives of New Zealanders. She refers to accept the reality of the impact of the virus and see the devastating effect, while behaving in terms of shopping in the “normal way.” She points out that responsible behavior is requested from each and every member of society and that single contacts can lead to the spread of the virus (Ardern, 2020).

Responsibility is highlighted by Ing-Wen (2020) in terms of the government responding to the pandemic by taking proactive measures to ensure a stable economy and financial stability. Ing-Wen (2020) further highlights that the government has also put a budget in please to prevent the spread of the disease. She also highlights her awareness about the concern about the legal basis for the restrictions put in place and the need for international cooperation.

#### Enjoyment and Evaluation

Merkel (2020) highlights that people should be creative to find new ways of entertaining and connecting, while Ardern (2020) points out that people can take walks and exercise outside on their own and with children. Ardern (2020) refers to the citizen making plans how to connect, letting the government enforce the new restrictions while having the citizen to “making phone trees” in the street and connecting to each other in kind and strong ways (Ardern, 2020).

Ing-Wen (2020) emphasizes: “The country deserves our confidence” and further highlights that people should refrain from negative feelings and behaviors and focus on the positive and accurate.

Based on this discussion Table 3 has been constructed.

**Table 3:** Global women leaders and the PURE model (Authors own construction).

**Leaders and PURE**	**Angela Merkel Germany**	**Jacinda Ardern New Zealand**	**Tsai Ing-Wen Taiwan**
Purpose	•Defeat virus and survive •Care for one another	•Slow down virus and save lives •Work together to support each other	•Fighting the virus by working together •Stand together and strengthen international cooperation
Understanding	•Deep understanding of the impact of this virus and the possibility of own approach and action	•Understanding how to stop the virus is easy, decisions to do so are not taken lightly.	•Understanding the situation, working together, refraining from negative feelings and behaviors share accurate knowledge to understand the situation
Responsibility	•Responsibility of each and every single individual: self-care and care for others	•Responsible action needs to be taken – Ardern does not differentiate between government and people •Stay home and stop interaction •Self-isolate if necessary, even when daunting	•Responsibility through governmental proactive measure on all levels •Awareness about the concern regarding the legal basis for the restrictions put in place and the need for international cooperation.
Enjoyment and evaluation	•Discipline to manage missing enjoyment through social connection and find new creative forms of entertainment	•Be practical and only leave home for walks, exercise and fresh air on your own and take children out.	•Be confident and refrain from negative feelings and behavior

## Conclusions and Recommendations

It is evident that Merkel (2020), Ardern (2020), and Ing-Wen (2020) in their efforts to reduce the impact of COVID-19 use their political leadership with particular emphasis on the leader-follower relationship, as well as selected aspects of PP2.0 and the PURE model. Through the speeches the women political leaders clearly and effectively communicate to the citizens the purpose and understanding of a shared focused vision based on the meaning of protecting lives and defeating COVID-19 nationally and internationally. The leaders implore the citizens to take up their responsibility to adhere to difficult strategies that should be implemented by authorizing them through drawing on their existing, shared skills and capacities. The three leaders also outline the different roles from which the citizens should fulfill particular subtasks tasks and in this way implore them to take up their shared responsibility for self-care and caring for each other by curbing the impact of COVID-19. With regards to aspects of PP 2.0 the three women emphasize the capabilities of the citizens to deal with difficult strategies and uncertainty within a specific period. Merkel (2020) and Ardern (2020) use different virtues of their citizens to call for self-care and care for each other national, whereas Ing-Wen (2020) refers to their virtues through a call for self-care and care within a national and international context. All three the political leaders use the meaning of each life and saving lives in their call to defeat COVID-19 through responsibly adhering to social distancing. The leaders also remind citizens that they have the resilience to deal with difficult, challenging strategies and interventions based on passed traumas and memories, as well as current actions. The leaders through creatively showing their personal capacity for caring, empathy and compassion for their followers, inadvertently connect and evoke their followers capacity for caring, empathy, compassion and general well-being to deal with physical and psychology vulnerability, uncertainty and turbulence. In this way the three women political leaders illustrate a fundamental assumption of PP2.0, i.e., that in maintaining well-being people should deal with the polarities of emotions that inherently carries positive and negative aspects (May, 2017; Mayer and May, 2019; Wong, 2019). In conjunction with this these political leaders have demonstrated the value of feminine protective characteristics, i.e., caring, empathy, compassion and creativity, as part of their wider leadership toolkit which we suggest include masculine protective characteristics. Perhaps these women are leading the way in showing that political leadership marked by the leader-follower relationship, include a broad variety of characteristics which are not gendered and enable (women) political leaders to transform pain, suffering, fear and despair into meaningful actions within times of Covid-19.

A possible limitation of this study could include that we chose the leaders and specific speeches which we appealed to us. This possible bias has been addressed by locating the findings within existing literature. Another critical point could be that we chose women leaders who are often perceived as “successful” in the eyes of the public. They might be classified as having “pre-existing success in their leadership”, even before Covid-19 and this might be one reason why they are successfully counteracting Covid-19. It is recommended that researchers to focus on other women and men political leaders to explore the nature an impact of their leadership beyond the confines of gendered leadership toward a more effective political leadership in times of crises which moves beyond stale ideologies about femininity and masculinity in leadership. Furthermore, it is imperative that these and other speeches be explored in more depth to understand the rational and irrational dynamics operating within the leader-follower relationship, as well as gendered leadership, for the purposes of enhancing the impact of all the effective characteristic of (political) leadership (during crises).

## Data Availability Statement

Publicly available datasets were analyzed in this study. This data can be found here: https://www.bundesregierung.de/breg-de/themen/coronavirus/statement-chancellor-1732296, https:// www.newshub.co.nz/home/politics/2020/03/coronavirus-prime-minister-jacinda-ardern-s-full-covid-19-speech.html, and https://english.president.gov.tw/News/5989.

## Ethics Statement

The researchers adhered to ethical considerations and standards while doing the literature review. Written informed consent for participation was not required for this study in accordance with the national legislation and the institutional requirements. Written informed consent was not obtained from the individual(s) for the publication of any potentially identifiable images or data included in this article.

## Author Contributions

All authors listed have made a substantial, direct and intellectual contribution to the work, and approved it for publication.

## Conflict of Interest

The authors declare that the research was conducted in the absence of any commercial or financial relationships that could be construed as a potential conflict of interest.
